# Advanced Multipurpose Spectroscopic Nanobio‐Device for Concurrent Lab‐on‐a‐Chip Label‐Free Separation and Detection of Extracellular Vesicles as Key‐Biomarkers for Point‐of‐Care Cardiovascular Disease Diagnostics

**DOI:** 10.1002/adhm.202500122

**Published:** 2025-06-02

**Authors:** Emma Buchan, Jonathan James Stanley Rickard, Mark Robert Thomas, Pola Goldberg Oppenheimer

**Affiliations:** ^1^ School of Chemical Engineering College of Engineering and Physical Science University of Birmingham Birmingham B15 2TT UK; ^2^ Department of Physics Cavendish Laboratory University of Cambridge JJ Thomson Avenue Cambridge CB3 0HE UK; ^3^ Institute of Cardiovascular Sciences College of Medical and Dental Sciences University of Birmingham Birmingham B15 2TT UK; ^4^ Healthcare Technologies Institute Institute of Translational Medicine Mindelsohn Way Birmingham B15 2TH UK

**Keywords:** cardiovascular diseases, extracellular vesicles, lab‐on‐a‐chip label‐free separation and detection, point‐of‐care diagnostics, spectroscopic nanobio‐device

## Abstract

The global aging population presents a major public health challenge, with cardiovascular diseases (CVDs) remaining the leading cause of death worldwide. Often asymptomatic in early‐stages, CVDs are frequently undiagnosed until critical events like myocardial infarction or stroke occur. To address this gap, an advanced integrated multipurpose spectroscopic lab‐on‐a‐chip bionano‐device has been developed for early CVD detection through extracellular vesicle (EV). EVs, which reflect the molecular state of their originating cells, are separated and analyzed using the combined Raman spectroscopy's molecular specificity with AI‐driven classification from clinical CVD biofluids. AIMSpec‐LoC unprecedently achieves rapid, label‐free, size‐based separation of EV subtypes, including small, mid and large EVs from biofluids, whilst preserving EV integrity and eliminating extensive preprocessing. The device enables real‐time, multiplexed molecular profiling of EV cargo, identifying CVD‐specific biomarkers with sensitivity and specificity >96% and linking these to CVD progression, achieving >97% accuracy in identifying disease‐specific molecular fingerprints. This bionanotechnological device generates quantitative barcodes to support prognostic modeling and therapeutic evaluation, providing clinicians with actionable insights for timely‐diagnosis and personalized treatment. AIMSpec‐LoC platform offers a transformative solution for point‐of‐care CVD diagnostics, addressing critical unmet needs in cardiovascular medicine, enhancing clinical decision‐making, improving patient health and reducing the global burden of CVDs.

## Graphical abstract

AIMSPec‐LoC is a novel lab‐on‐a‐chip platform integrating size‐based extracellular vesicle (EVs) separation with *label‐free* Raman spectroscopy and AI‐powered classification via SKiNET. This high‐throughput, portable system enables real‐time, multiplexed molecular fingerprinting of EVs from biofluids, offering transformative potential for early, non‐invasive cardiovascular disease diagnostics and personalized clinical decision‐making at the point of care.

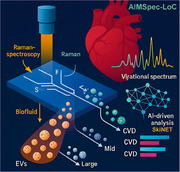

## 1 Introduction

Cardiovascular diseases are the leading cause of morbidity and mortality worldwide, with high‐complication rates. In 2019, 20.5 million people died from CVDs, accounting for 32% of global deaths.^[^ ^1^ ^]^ Although, many CVDs may be prevented by addressing behavioral risk factors for heart disease and stroke, such as physical inactivity and tobacco use, no underlying atherosclerotic arterial disease has yet been identified and only the fatal cardiac events including stroke or heart attack, often constitute the first signs of disease. The inefficient prevention of CVDs increases the incidence of cardiovascular‐related health conditions, contributing to higher demands on the healthcare system. Furthermore, the steadily growing elderly population increases the demand from the health and social services for prolonged periods as patients with multiple CVDs require long‐term management with repeated visits to healthcare professionals. Therefore, it is crucial to detect CVDs as early as possible to reduce the major burden on the healthcare systems through the inclusion of management, interventions and medications.

Quantitative measurement of a patient's disease state via condition specific biomarkers is crucial in modern practice to guide tactful therapeutic treatment.^[^ ^2^, ^3^ ^]^ CVD‐indicative biomarkers can provide insights into the biological pathways underpinning certain pathologies allowing effective stratification and classification of pathology for the purpose of guiding management.^[^ ^3^, ^4^, ^5^ ^]^ However, typical time‐to‐results of many diagnostic and prognostic tests specific to CVDs such as electrocardiogram, CT coronary angiography, MRI and radionucleotide tests are often greater than a month^[^ ^6^ ^]^ These tests are expensive and delayed access provides a barrier to initiation of effective preventive treatments, leading to avoidable mortality. Conventional diagnostic methods often involve invasive procedures such as, coronary angiography in CVD, are associated with small risks of heart attack, stroke, infection and discomfort for patients.^[^ ^7^ ^]^ Thus, there is a pressing need for rapid, reliable, non‐invasive diagnostic approaches. The development of a disposable, simple and low‐cost bedside device to measure potential biomarkers of early CVD, from readily available biofluids such as, blood‐plasma or saliva, would be of vital importance and a major step toward the determination of an individual's disease state. It would also allow for the accurate measurement of the efficacy of specific clinical therapies and interventions, indicating how an individual is clinically progressing.

In recent years, extracellular vesicles have been emerging as promising circulating biomarkers of disease.^[^ ^8^, ^9^ ^]^ EVs are lipid bound membranes, released by cells into the extracellular space. They are present in most body fluids, including blood, urine and saliva, and therefore, are easily accessible for analysis.^[^ ^10^, ^11^, ^12^, ^13^ ^]^ EVs are considered as reservoirs of important information as they hold key evidence relating to the cells from which they originate. Acting as intracellular messengers, their role in the potential spread of disease has further garnered increasing interest.^[^ ^14^, ^15^, ^16^, ^17^ ^]^ Although still largely unknown, there are multiple routes for generation of EVs, with subpopulations divided into three broad groups based on their size, density and composition, which typically include exosomes, microvesicles and apoptotic bodies.^[^ ^18^, ^19^ ^]^ Exosomes form as a result of the inward budding of endosomal membranes inducing the formation of multivesicular bodies (MVBs). MVBs are subsequently released into the extracellular environment by fusion with the plasma membrane.^[^ ^20^, ^21^ ^]^ Upon their release into either the parenchymal space or the urinary space, exosomes can participate in molecular signalling events.^[^ ^22^ ^]^ Microvesicles, on the other hand, are formed by direct budding from the plasma membrane.^[^ ^23^ ^]^ Their formation is induced in the presence of a stimulus such as, oxidative stress, which drives cellular events, for example, phospholipid‐binding proteins. This in turn, leads to the budding and release of microvesicles from plasma.^[^ ^24^ ^]^ Apoptotic bodies are released during the latter stages of cell death and contain a multitude of cellular organelles, cytosolic content and nuclear materials.^[^ ^25^ ^]^ Despite each EV subgroup exhibiting apparent differences in their mechanisms of formation, once secreted from a cell, it is difficult to discern one EV subtype from another. These vesicles do not contain a specific molecular marker or descriptive property distinguishing the sub‐groups.^[^ ^26^ ^]^ The overall potential as key biomarkers, vaccines and drug delivery vessels has attracted a growing interest in EVs, which carry and protect cellular cargoes, including nucleic acids, proteins and lipids from their place of origin and thus, reflect the physiological and pathological state of their parent cells.^[^ ^27^, ^28^, ^29^ ^]^ The detection and analysis of EVs therefore, offers valuable insights into the disease mechanisms, diagnosis, prognosis as well as response to therapeutics.

EVs are heterogeneous in origin, molecular constituents and size, and whilst they are present in an array of complex biofluids, these comprise differing amounts of non‐vesicular structures, which may impede analytical results.^[^ ^27^ ^]^ The isolation and separation of EVs is therefore a required pre‐analytical step. Conventional methods including, for instance, ultracentrifugation, size exclusion chromatography (SEC) and immunoaffinity approaches used to investigate EVs suffer from low throughput times and require expensive equipment, highlighting the increasing demand for improved analytical methods.^[^ ^30^, ^31^, ^32^, ^33^, ^34^ ^]^ Microfluidics has recently been emerging as an additional technique in EV research^[^ ^35^, ^36^, ^37^ ^]^ with the lab‐on‐a‐chip (LoC) based technologies holding promise for overcoming many of the challenges associated with existing EV isolation methods. By leveraging microfluidic systems, LoC devices can provide enhanced control over fluid flow, enabling precise and efficient isolation of EVs.^[^ ^38^ ^]^ The miniaturized nature of these devices allows for reduced volumes, rendering them suitable for time‐sensitive or limited sample sources. Moreover, LoC platforms offer added opportunities for automation, standardization and multiplexed processing, leading to improved reproducibility and scalability of EV isolation protocols.^[^ ^39^ ^]^ The miniaturisation and integration of quantitative analytical techniques within the chip, such as molecular profiling methods via Raman spectroscopy (RS), can enable the further facilitation of the downstream rapid, non‐invasive and accurate analysis of isolated EVs.

Raman spectroscopy is a sensitive analytical method, which provides a unique spectral fingerprint of target analytes via the inelastic scattering of light. It enables non‐destructive quantitative analysis of biochemical composition, requires no complex sample preparation and poses an inherently straightforward detection in aqueous conditions. Furthermore, its portability enables reliable in‐vivo point‐of‐care diagnostics. Raman spectroscopy technology could therefore, offer a label free, non‐invasive route for measuring changes in CVD biochemistry at the earliest stages of disease with a straightforward integration into existing healthcare pathways^[^ ^40^, ^41^ ^]^ This non‐invasive and label‐free analysis of EVs provides detailed molecular information of their composition and structural properties with further advantages including the elimination of the need for exogenous dyes or labels, which could alter the EV cargo or introduce artifacts as well as providing high molecular specificity, enabling rapid identification and quantification of various biomolecules, for example, proteins, lipids, nucleic acids and metabolites within EVs.^[^ ^42^, ^43^, ^44^ ^]^ Through close examination of spectroscopic bands associated with specific molecular vibrations, RS discriminates between molecular species and provides insights into their relative abundance as well as information regarding the secondary structure of proteins, confirmation of nucleic acids and the ordering of lipids within the EV membranes, offering overall valuable insights into vesicle composition and organization.^[^ ^45^, ^46^ ^]^ The integration of RS with LoC technology for EV separation, detection and analysis therefore has a significant potential to enable the efficient and high‐throughput isolation along with a controlled and reproducible environment for analysis, whilst performing real‐time and in situ detection of isolated EVs, yields rapid characterisation of their molecular composition.

Due to the challenges associated with EV characterisation and classification using conventional analytical techniques, as well as the high‐dimensional datasets generated through techniques such as flow cytometry, omics profiling, and RS, machine learning (ML) and artificial intelligence (AI) have emerged as powerful solutions to overcome these limitations.^[^ ^47^ ^]^ A variety of ML algorithms have been applied to EV research, including supervised models such as support vector machines (SVM), logistic regression, linear discriminant analysis (LDA) and neural networks which can be trained to classify EV subtypes and/or predict disease states.^[^ ^48^, ^49^, ^50^, ^51^ ^]^ Wang *et* al. for example, combined LDA analysis with electrochemistry for the analysis of tumour EVs, where using an integrated microfluidic electrochemical device and detecting low abundance targets, the authors were able to accurately identify breast cancer patients with 100% sensitivity and specificity.^[^ ^52^ ^]^ In addition, Li *et* al. applied logistic regression and SVM in the diagnosis of non‐small cell lung cancer (NSCLC), achieving diagnostic accuracies of 97.4% for the training cohort and 91.7% for the validation cohort when detecting the expression of epidermal growth factor receptor (EGFR) and C‐X‐C chemokine receptor 4 (CXCR4) in serum EVs as indicators and predictors of NSCLC.^[^ ^51^ ^]^ Pulliam et al. also applied SVM to predict HIV‐associated neurocognitive disorder in HIV‐infected people using plasma neuronal EV proteins with an area under the curve value of 0.82 and Lee *et* al. used various ML methods including logistic regression, random forest, SVM and Deep Neural Network (DNN) to obtain candidate markers in genus and phylum levels linked to the diagnosis of pancreatic cancer from blood derived EVs with DNN using 11 outperforming other ML methods with an area under the curve of 0.96.^[^ ^53^, ^54^ ^]^ Similarly, unsupervised methods such as k‐means clustering, hierarchical clustering, and principal component analysis (PCA) have also been applied with varying degrees of success in the exploration of EV heterogeneity and to reveal intrinsic patterns within complex EV datasets.^[^ ^48^, ^49^, ^50^, ^55^ ^]^ Shin *et* al. for example, identified Raman patterns associated with normal and cancerous exosomes using PCA and Dobra et al. applied PCA to determine sEVs were more suitable to discriminate between patients diagnosed with glioblastoma multiforme, non‐small‐cell lung cancer brain metastasis, meningioma, and lumbar disc hernia patients.^[^ ^50^, ^56^ ^]^ The integration of computational approaches not only improves diagnostic accuracy and allows deeper insights into EV biology but also pushes the field toward more robust and clinically applicable solutions.

Herein, we have developed a portable advanced integrated multipurpose spectroscopic lab‐on‐a‐chip for isolation, detection and analyses of EVs via specific RS fingerprinting for low‐cost, non‐invasive, early‐stage diagnosis. Design and development of our unique optofluidic Raman lab‐on‐a‐chip device technology for detection of CVD‐indicative biomarkers enables timely detection of EVs as key‐disease indicators, as a platform toward providing transformative triaging and diagnostics. AIMSPec‐LoC rapidly extracts EVs from biofluids and detects these as key‐markers with high‐sensitivity, specificity and timeliness. The developed lab‐on‐a‐chip technology combined with Raman spectroscopy subsequently, is validated for the detection of cardiovascular and inflammatory bowel disease indicators, establishing a powerful analytical tool for biomolecular analysis. The acquired spectral data via AIMSPec‐LoC are subsequently classified using our advanced artificial neural networks self‐optimizing Kohonen index network (SKiNET) algorithm as a decision support tool for easy readouts for clinicians. SKiNET is based on self‐organizing map (SOM) with a classification via the self‐organizing map discriminant index (SOMDI). Through inspection of key differences between neuron weights and class weight vectors, the algorithm enables identification of the key spectral changes. These allow the identification of the types of data a given neuron activates, which are used to inspect the weights across all neurons and extract prominent features belonging to each class by finding the weights that contribute most to a particular class. The peaks in SOMDI subsequently correspond to cm^−1^ and modes that contribute most to the clustering observed in the SOM. Training parameters used for the SOM include grid size, learning rate and optimal number of epochs and the separation of classes reveals the characteristic differences due to the classification of certain neurons. This enables a clear basis for differentiation via the characteristic weight vectors to be derived in SOMDI. In this study, SKiNET enabled accurate classification of EV subtypes and disease states, achieving high accuracy for small EVs, while also visually mapping disease progression via spectral barcoding. These performance metrics exceed those reported by most label‐based or electrochemical ML systems. Our platform also offers greater flexibility for real‐time diagnostics and scalable point‐of‐care use, owing to its compact form factor, minimal sample processing and high‐throughput capacity. Overall, the integration of AIMSPec‐LoC with SKiNET delivers a powerful, label‐free and multiplexed diagnostic solution, advancing beyond current state‐of‐the‐art ML‐assisted EV detection platforms in terms of resolution, robustness, clinical relevance and practical deployability. AIMSPec‐LoC is further shown to overcome several of the challenges associated with existing methodologies for the isolation of EVs. For instance, field‐flow fractionation (FFF) used in EV isolation, where a sample is injected into a chamber is often affected by a cross flow with smaller particles eluting earlier,^[^ ^57^ ^]^ takes 1 h to perform, requires significant sample pre‐preparation to remove larger cells and debris and can only be operated by highly trained specialists. In contrast, AIMSPec‐LoC requires no sample pre‐preparation and analyses biofluids in their native state. A further isolation technique commonly applied in microfluidic EV studies is immunoaffinity capture (IAC). IAC relies on the antigenic identification of EV surface components (primarily proteins) and although is rapid and selective when used for either preparation or analysis, one intrinsic drawback of this technique is the requirement for prior knowledge of the surface antigen to be targeted, and which, may be present across multiple subpopulations of EVs.^[^ ^58^ ^]^ Targeting only a single antigen is further exacerbated by the loss of potentially important EV populations.

Overall, by leveraging the ability of RS to analyze complex molecular signatures, we have identified disease specific EV markers associated with CVD. Through a comprehensive analysis combined with advanced computational algorithm, our developed novel approach for the accurate and early detection of CVD, ultimately would lead to improved patient outcomes and more effective disease management. Measuring abnormal changes in specific biomarkers would be indicative of CVDs, providing a quantitative assessment at the earliest stages whilst simultaneously helping to quantify the damage. It would be interpreted by clinicians as an indication to initiate treatment before advanced disease has developed. In the long‐term, this platform will be extended to further applications, for example, early‐recognition of neurodegenerative diseases, degeneration and timely interventions specifically targeting the identification of CVDs in otherwise healthy individuals. The integration of simultaneous EVs isolation with Raman spectroscopy holds great promise for disease diagnostics. It can improve the discovery of future cardiovascular markers for early‐diagnostics and intervention as well as advance the understanding, prevention and treatment of CVDs and the associated detrimental conditions.

## 2 Results and Discussion

**Figure 1:**
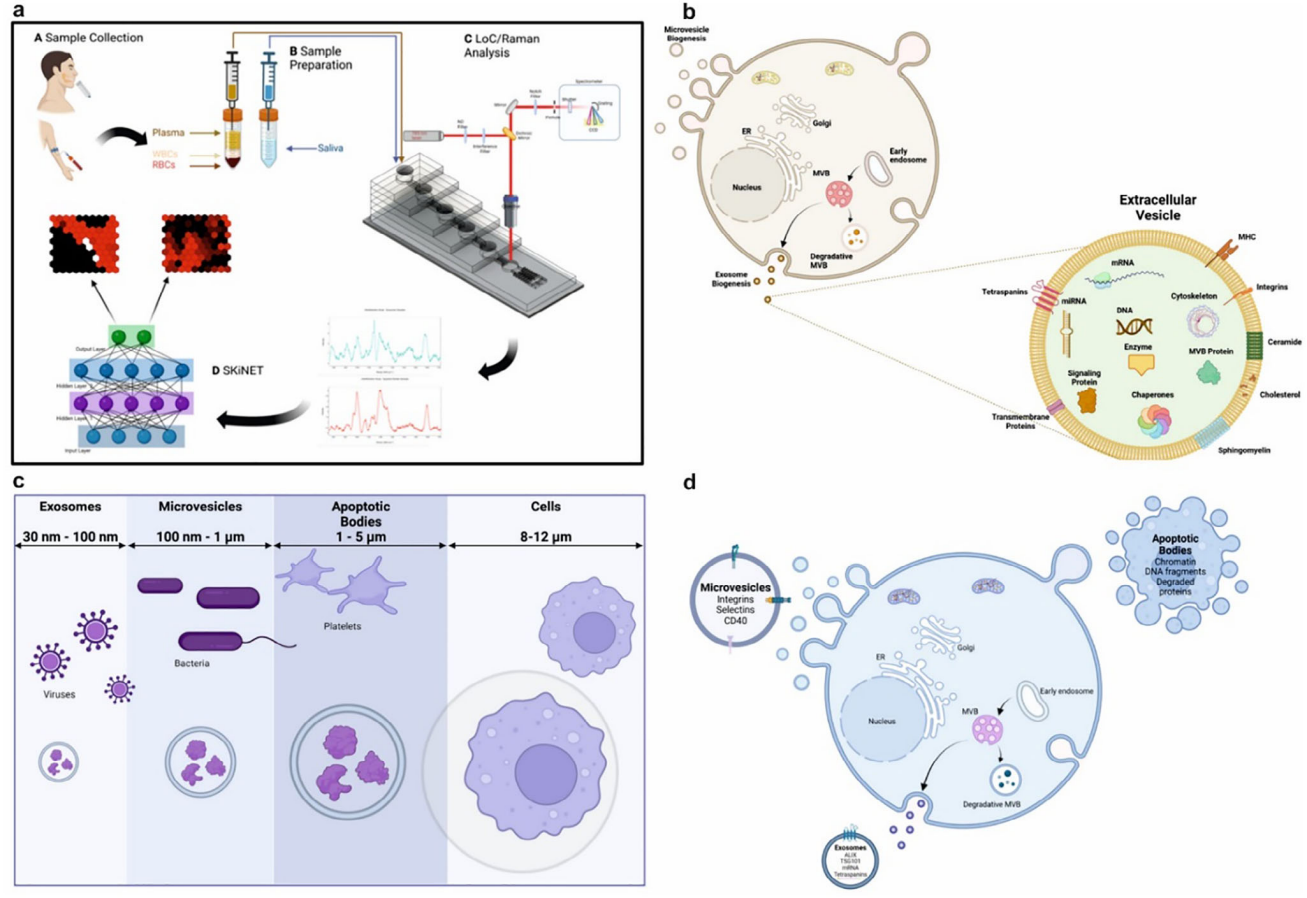
Overview of the EVs Diagnostic Principles. a). Schematics of LoC methodology including the sample collection from biofluids, for example, saliva and blood, followed by sample preparation to isolate plasma via centrifugation and dilution of saliva with on‐chip isolation, separation and the subsequent Raman detection of EVs obtained from saliva and blood plasma and rapid analysis via artificial neural network algorithm, SKiNET, acting as a decision support tool in the classification of acquired data, output in both the diseased state as well as subgroup of isolated EVs (D). b). EV biogenesis illustrating the formation of exosomes via the inward budding of multivesicular bodies (MVBs) within the cell, resulting in the release of intraluminal vesicles as exosomes when MVBs fuse with the cell membrane and microvesicle formation through the direct outward budding or shedding of the plasma membrane. Composition and structure of EV illustrating phospholipid bilayer surrounding protein (signalling protein, MVB protein) and nucleic acids (DNA and RNA). Membrane proteins including tetraspanins, MHC, receptors, and adhesion molecules (integrins). c). Typical size ranges of the three main EV subgroups compared with cells. Apoptotic bodies range from 1–5 µm, smaller microvesicles range from 100 to 1000 nm, overlapping with bacteria and exosomes are commonly defined as vesicles with sizes in the range of 30–100 nm. d). EV formation highlighting the main contents and surface markers of each vesicle type. Exosomes contain ALIX, TSG101, mRNA and tetraspanins whilst integrins, selectins and CD40 are characteristic of microvesicles and apoptotic bodies are primarily composed of chromatin, DNA fragments and degraded proteins.

A unique lab‐on‐a‐chip device capable of isolating and separating extracellular vesicles as key targets of disease has been developed, offering highly versatile capabilities with faster analysis times compared to conventional methods such as ultracentrifugation and subsequently validated for cardiovascular and inflammatory bowel disease identification. The miniaturized design, eliminates the need for multiple separation steps, reduces the required sample volume and enables the integration of isolation and downstream analysis onto a single platform (**Figure** **1**a). The portability and compactness of the LoC is well suited for point‐of‐care applications, allowing for their deployment in various clinical to provide real‐time diagnostics.

The AIMSpec LoC was designed and engineered to enable sufficient isolation of various populations of EVs, including small, mid and large EVs within size ranges of 40–100, 101–200, 201–500, and 501–5000 nm, respectively. In addition, the platform was designed to facilitate isolation and separation of up to 1 ± 0.3 mL of sample at final concentrations ranging from 1 × 10^6^–9 × 10^8^ EVs from an array of biofluids, while simultaneously allowing the separation of EVs into respective size‐based subgroups. The unprecedented, generated ability of the LoC to analyze various vesicles in parallel from multiple body‐fluid sources provides important and valuable insights into the tissue‐specific release of EVs and their potential roles in different diseases and biological processes. Figure 1 provides a comprehensive overview of the experimental setup, illustrating the integration of the designed LoC, the biogenesis of EVs with the key components of the methodological workflow (Figure 1a) including, the sample collection, on‐chip isolation, separation and subsequent Raman analysis with an AI algorithm data classification, all of which were designed and engineered to achieve optimal outputs of speed, specificity and accuracy. Simultaneously, it highlights the cellular processes involved in the biogenesis of different EV subgroups, for example, the outward budding of EVs from the plasma membrane and their release into the extracellular space (Figure 1b) as well as representative size‐based differences of each group (Figure 1c). Typically, micro‐platforms focus on the isolation of specific EV subtypes, either exosomes or microvesicles and incorporate capture mechanisms based on immunoaffinity or surface‐marker selection. Here, we have uniquely fabricated LoC device capable of a broad‐spectrum EV isolation, designed to capture a wide range of subtypes, collectively enabling not only a comprehensive analysis of the EV population present in the sample but also, allowing for a more inclusive and unbiased characterisation of EVs and their cargo.

**Figure 2:**
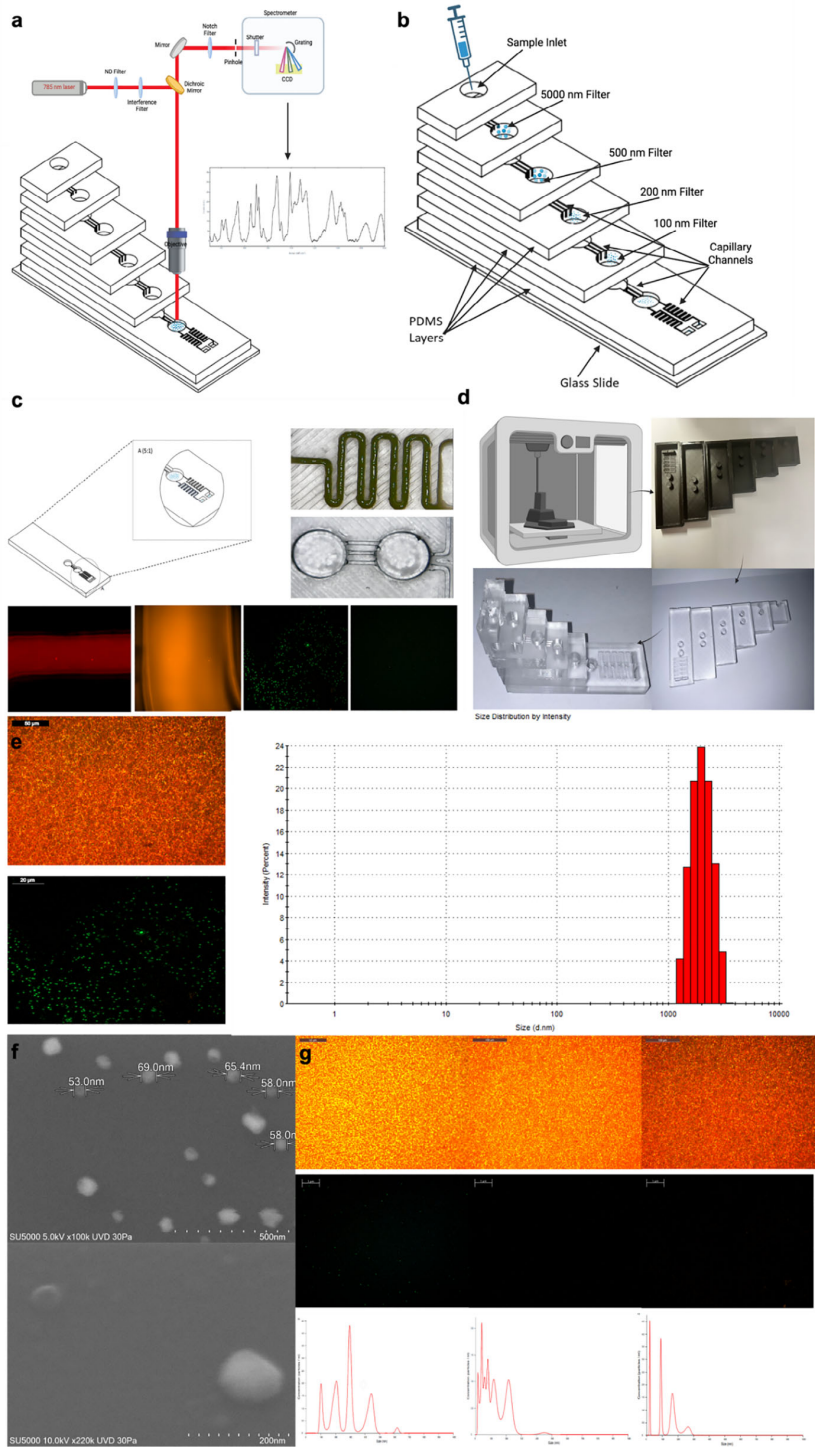
Fabrication and Characterisation of AIMSPec‐LoC for the Isolation and Separation of EVs. a). Schematic illustrating the overall chip design complete with integrated downstream analytical EV Raman analysis. b). Detailed annotated diagram of the LoC showing the sample inlet in the first PDMS layer with the 5000, 500, 200, 100, and 40 nm filters in subsequent layers and the capillary channels, aiding fluid flow within the system. c). Schematics of the capillary flow within the LoC base layer (left) with optical imaging of capillary channels (top right) and microfluidic channels (bottom right) connecting sample inlet and sampling well. d). Overview of the LoC fabrication including, 3D printing (top left), PDMS casting (top tight and bottom right) and the 3D‐assembly (bottom left), yielding the prototype AIMSPec‐LoC model. **(e)**. Optical microscopy image (top left) of the 500 nm pore size nanoporous filter with the corresponding fluorescence microscopy image (left bottom) of collected 500–5000 nm EVs and DLS measured via Panalytical Zetasizer HPPS (right hand side) exhibiting abundance of nanovesicles in the range of 1000–5000 nm. f). SEM images of the 100 nm nanoporous membrane filter indicative EVs and representative SEM image of isolated EVs. g). Fluorescence microscopy images of the movement of fluorescent beads, acting as visual proxies for EVs within the microfluidic channels and their associated collection in the sampling well, demonstrating the separation efficiency of the LoC and nanoparticle tracking analysis measured (NanoSight N300) demonstrated abundance and size distribution of isolated EVs for each filter size.

The LoC is designed using a polyethersulfone nanoporous membrane based on a size‐based exclusion and filtration approach (**Figure** **2**), fabricated to separate EVs from biofluids, within which a filtered plasma is acquired from 50 µL of blood in <10 minutes with a similar performance enabled for the filtration of 50 µL saliva (1:2v/v in PBS) in <15 minutes. Comprehensive characterisation of the AIMSPec‐LoC, essential in the assessment of chip performance and its suitability for the efficient isolation and separation of EVs, incorporated multiple techniques including, nanoparticle tracking analysis, optical microscopy, fluorescence microscopy and dynamic light scattering, in accordance with the international society for extracellular vesicles recommendations of at least two characterisation methods to be used for EVs. The combined use of these analytical techniques provides a systematic evaluation of the LoC functionality, EV size distribution and captures efficiency as well as highlights the robustness and an overall performance of the chip in EV isolation and separation, providing further insights for its potential straightforward integration into existing healthcare pathways as a diagnostic modality. The engineered LoC consists of six adhered PDMS layers, with the top layer serving as the primary sample inlet (Figure 2a–d). Each subsequent layer consists of a further sample inlet and a sample collection well. The PES membranes, of varying sizes (40, 100, 250, 500, and 5000 nm) act as filters placed between each PDMS layer at the base of the sample collection wells. Capillary channels (0.05 × 0.05mm^2^) are placed in the base layer to aid fluid flow through the chip with the porous membranes in constant fluid contact. Whilst particles or EVs larger than the pore size are unable to pass through the membrane and trapped in their respective sample well, smaller particles / EVs pass through the membrane due to the faster diffusion. An additional membrane (pore size 40 nm) is included in the design to trap proteins and reduce contamination (Figure 2d). The overall membrane placements and collection wells were designed to maximize the recovery of samples. The size distribution and concentration of EVs captured via the engineered LoC has been subsequently analyzed via NTA whilst leveraging the Brownian motion of the particles in solution (59) The representative size distribution profile of EVs isolated has been found to be in three main ranges of 41–100, 101–250, and 251–500 nm, indicative of small, mid and large EVs, respectively (Figure 2j). Corresponding well‐defined size distributions with mean diameter of 61 ± 23, 189 ± 84, and 432 ± 143 nm, respectively, indicate a successful isolation of EVs within the desired ranges.^[^ ^22^, ^24^ ^]^ The average EV concentrations were found to be 7.7 × 10^8^ and 2.2 × 10^8^ particles mL^−1^ in each isolated sample well, confirming the efficient capture capabilities of the pore size‐based separation of plasma based EVs, indicative of CVD. Further, size distribution of the larger isolated particles determined via DLS reveals distribution profile of 501–5000 nm with a population consistent with the large EVs^[^ ^25^ ^]^ (Figure 2h).

To further assess and evaluate the specific size‐based capturing of the EVs, fluorescent polystyrene beads ranging from 5 µm to 30 nm were used to represent the EVs dimensions for each studied subgroup, that is, 1 and 5 µm for large EVs, 0.2, 0.45, and 0.5 µm for mid EVs and 0.04 and 0.1 µm to for small EVs. Representative fluorescence microscopy images of the size‐based distribution show the fluorescent particles flowing through the microfluidic channels (Figure 1,i–j) exhibiting intense fluorescent signal corresponding to the specific sample labelling, that is, PS beads in the range of 41–100 (red), 101–250 (blue), and 251–500 nm (green). This indicates specific capturing of the target ranges with negligible sample signal crossover and an overall separation efficiency of 92% (calculated from the total number of beads collected across all filtration membranes divided by the number of those gathered from a single filter membrane), demonstrating the capability of the LoC to selectively capture size based EVs, delivering a robust platform for downstream analysis and rapid molecular profiling. Optical microscopy images of the nanoporous membrane structure (Figure 2j) showing the surface and internal features to determine pore size, shape and distribution, further enable identification of defects or irregularities as well as real‐time monitoring to eliminate blockages. SEM images of the EVs in solution, captured on surface of the nanoporous membrane from each of the filtration steps (Figure 2k), further confirm the EV subgroups isolated with size ranges of 40–100 and 101–200 nm and average EV sizes of 61 ± 3 and 189 ± 84 nm, respectively, in correspondence with the NTA analysis.

Important to note, the fabricated chip enables the label‐free isolation of EVs and their associated subgroups via size‐based and capillary functions by applying *g*‐force to the bioparticles, which is several orders of magnitude lower than the typical forces applied in conventional ultracentrifugation, reducing the overall particle aggregation and damage due to shear stress. The LoC, as a passive cataloguing technique, exhibits further advantages over traditional methods such as ultracentrifugation and immunocapture, including the overall lower cost of the system, requiring no additional external force fields in contrast to syringe pumps routinely used in EV microfluidic systems,^[^ ^35^ ^]^ nor does it require antibodies to capture the EVs. The cost‐effective engineered design is based on gravity forces combined with capillary channels, enabling high throughput fluid flow through the LoC, yielding a smaller, more compact chip than other microfluidic devices.^[^ ^60^, ^61^ ^]^ Typical blockage issues, often associated with microfluidic systems as well as channel deformations are avoided within the AIMSPec‐LoC due to the larger channel dimensions and overall lower flow rate. The ability to isolate EVs directly from saliva and blood plasma offers several key benefits for use in clinic or at bedside including, rapid time‐to‐results (<20 min), portability (75 × 25 × 50mm^3^) and high accuracy (AUC of 0.95‐1). It also enables ease of deployability in remote or resource limited settings for timely diagnosis and treatment even in areas with limited access to laboratory equipment, automation and minimal user intervention reducing the potential human error, lower cost per test with reduced consumption of reagents as well as inherent scalability for future mass production

Finally, the unique ability of the designed LoC to isolate EVs from various biofluids, such as saliva and blood plasma, enables multiplexed assay detection. Whilst the microfluidic devices typically are applicable for a single biofluid for the EVs capture,^[^ ^61^, ^62^, ^63^ ^]^ AIMSPec‐LoC platform is suitable for simultaneous multi‐biofluid EV capture and isolation, to provide a superior overview of a patient's health status whilst reducing the requirement for multiple costly tests.

The overall separation efficiency of the LoC of 92 ± 6%, confirmed by fluorescence microscopy combined with NTA (Figure 2g,i) is found to be significantly higher than size exclusion chromatography of 67.7 ± 13.1% as well as than the polymer‐based precipitation ≈82%.^[^ ^64^ ^]^ A *t*‐test confirmed statistically significant separation (p**<0.01) between EV populations collected from different channels. This separation efficiency can be further increased following dilution (1:10) of the tested biofluids however, this would be at the expense of the throughput and a proportional reduction in the final sample EV concentration for downstream analysis. Our AIMSPec‐LoC efficiently processes up to 500 µL of saliva within 20 minutes (≈1.5 mL h^−1^), increasing to 650 µL (≈1.95 mL h^−1^) for blood plasma (Figures S1 and S2, Supporting Information).

Our system does not rely on any external forces for EV isolation^[^ ^65^ ^]^ and has a unique ability to simultaneously isolate different size groups of large, mid and small EVs in a single step. This contrasts with “gold‐standard” of ultracentrifugation for EV isolation, which is uncapable to separate several EV groups concurrently and requires further density gradient to reach higher purification.^[^ ^30^ ^]^ Furthermore, to tackle the common issue with EV isolation of the co‐elution contaminating lipoproteins and plasma proteins,^[^ ^66^ ^]^ we employed low protein binding PES filter membranes, successfully trapping the contaminating proteins via the 40 nm filter as well as the lipoproteins including LDL with a size range of 25–35 nm. Whilst traditional techniques such as size exclusion chromatography (SEC) are effective at removing proteins, they are unable to efficiently remove lipoproteins, requiring density gradient ultracentrifugation to aid in the exclusion of these contaminants and thus, the combined approach suffers from significant losses of EVs.^[^ ^67^, ^68^ ^]^ In the AIMSPec‐LoC, the hybrid isolation and spectroscopic detection enable the multiplexed ability whilst removing any signal associated with contaminating proteins or lipoproteins. Furthermore, size‐exclusion methods often suffer from clogging and trapping in the membrane, poor efficiency and deformation of EVs due to pre membrane pressure.^[^ ^69^, ^70^ ^]^ These drawbacks are circumvented in the AIMSPec‐LoC via the introduction of sequential filtration with the initial step using a large pore filter (5000 nm), followed by sequential filtration steps through smaller pore filters (450, 200, 100, and 40 nm). Additionally, removing the need for external syringe or centrifugal pumps and including capillary channels to propel the flow through the system overcomes pressure associated destruction of EVs.

Identifying and characterizing different types of EVs can be challenging due to several factors including the small sizes (with overlaps between the two main types of EVs, microvesicles and exosomes), heterogeneity and the lack of standardized isolation and characterisation methods. Therefore, the identification of biomarkers associated with EVs can significantly aid in their identification and characterisation. These can provide specific differentiating characteristics of EVs from other particles or contaminants in biological matrices since specific proteins, lipids, or nucleic acids serve as unique markers for different EV types (e.g., CD9, CD63, CD82). Since EV subgroups exhibit distinct biomarker profiles, identifying these biomarkers enables the classification of EVs whilst providing valuable information on their cellular origin, biogenesis and functional roles. In addition, changes in the composition of EV biomarkers are associated with various diseases. Identification of these would further have diagnostic, prognostic as well as therapeutic implications, with the current research focused on exploring the potential exploitation of the latter for early detection and monitoring of diseases such as cancer, neurodegenerative disorders and cardiovascular diseases. To further validate the isolation of EV subgroups, a panel of known EV biomarkers was analyzed (Figures S3 and S4, Supporting Information) to acquire the Raman spectral fingerprints and compare their signatures with the EVs isolated and separated via the AIMSPec‐LoC.

Microengineered AIMSPec‐LoC has been subsequently validated via analysing clinically relevant samples. Biochemical fingerprints of EVs derived from blood plasma and saliva correlating with CVD were investigated via Raman spectroscopy. Through the subsequent SKiNET examination of their spectral shift, generated from the molecular vibration frequencies of specific molecules including amino, fatty and nucleic acids, the disease state of the patients has been determined. The clinical blood plasma was collected as part of the CASCADE study (Ethics Ref. 19/SW/0010), providing samples from CVD and healthy control subjects (**Table** **1**) with the consequent EVs collected from the LoC directly detected and analyzed via Raman spectroscopy (10 × 10µm^2^ sample area, 785 nm laser). Overall, a total of 4000 measurements were collected from blood plasma samples of CVD group (*n* = 20), 20 healthy volunteers acting as a control group (Methods).

**Table 1:** Demographics comparing epidemiological and clinical parameters of CVD patients.

	Control (*n* = 20)	CVD (*n* = 20)
Age (years)	48.5 ± 11.5	65.5 ± 23.5
Sex (Male/Female)	28/12	49/11
Systolic blood pressure (mmHg)	122 ± 8	135 ± 25
Diastolic blood pressure (mmHg)	77 ± 9	84 ± 36
BMI (kg m^−2^)	28.2 ± 4.2	34.3 ± 6.8
Presentation (*n*) STEMI NSTEMI Atrial Fibrillation	‐ ‐ ‐	10 (50%) 6 (30%) 4 (20%)
Medication (*n*) Aspirin Clopidogrel Ticagrelor	0 0 0	18 (90%) 10 (50%) 14 (70%)

**Figure 3:**
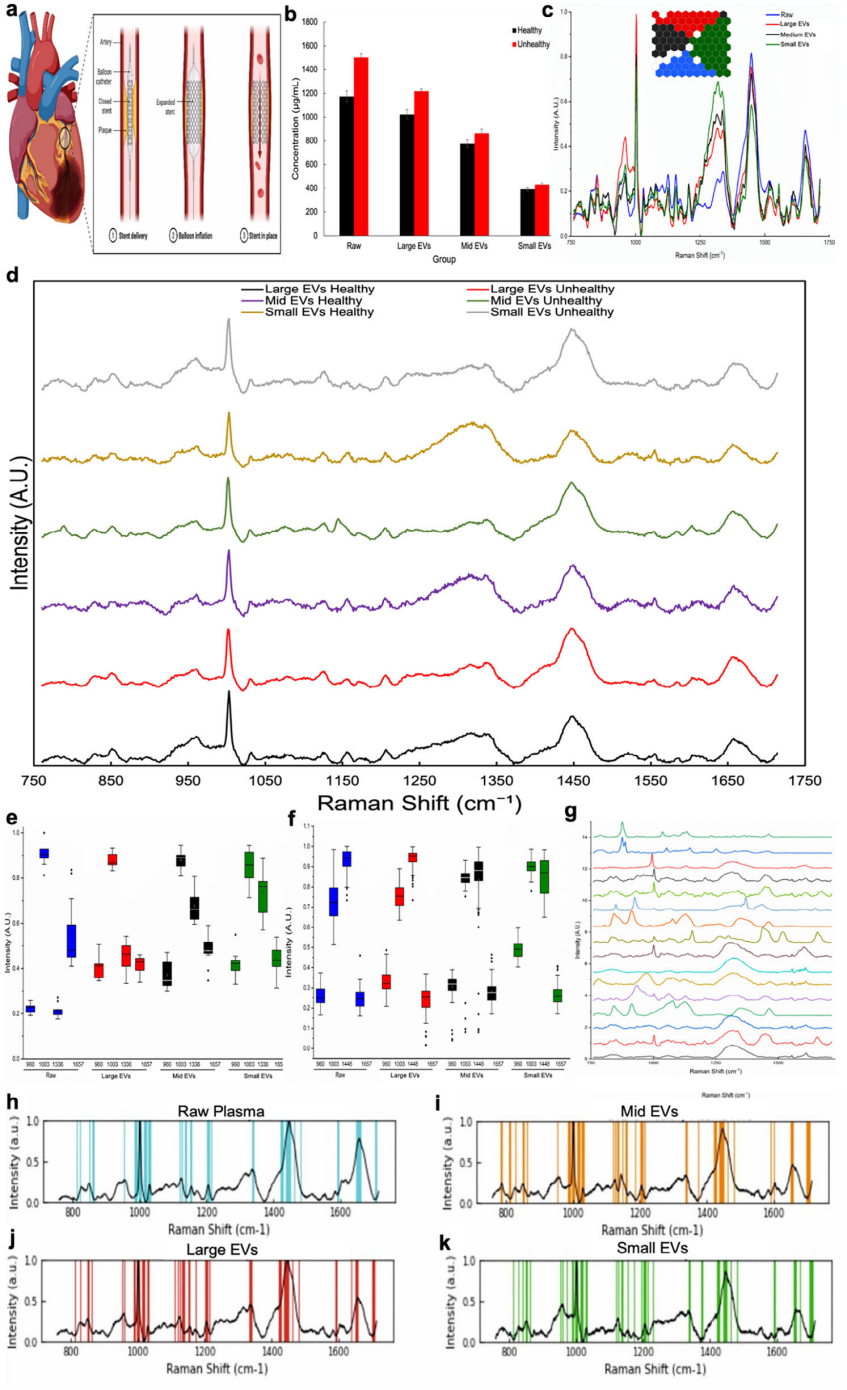
Spectral Detection and Differentiation of Cardiovascular Disease from Blood Plasma EVs. a). Illustration of damage sustained to the heart after myocardial infarction with typical stenting procedure shown. Coronary angiogram **(inset)** typically used for clinical CVD diagnosis. b). BCA protein assay indicating differences in protein concentration at each filtration step indicative of 3 main EV subgroups: large EVs, mid EVs, small EVs and raw blood plasma. c). SOM (inset)/SOMDI of EVs classified with an accuracy of 96.5 ± 0.4%. Sub classification according to EV subgroup are represented by blue hexagons for raw blood plasma, red for large EVs, black for mid EVs and red for small EVs. Representative average Raman spectra of EV subgroups of healthy and CVD (unhealthy) of d) large EVs, mid EVs and small EVs.(e,f). Box and whisker plots represent the minima, maxima, interquartile ranges, whiskers and the median in peak intensities identified via SOMDI as having the greatest effect on the classifier, that is, the spectral changes of greatest importance with the largest changes observed of 851, 960, 1003 and 1657cm^−1^. g) Representative Raman spectral fingerprints of a range of CVD‐indicative biomarkers of interest, including apolipoprotein B (ApoB), lipoprotein A (LpA), proprotein convertase subtilisin/kexin type 9 (PSCK9), C‐reactive protein (CRP), d‐dimer, interleukin‐9 (IL‐9), myeloperoxidase (MPO), tumour necrosis factor‐alpha (TNF‐α), copeptin, galectin‐3, growth and differentiation factor‐15 (GDF‐15), matrix metalloproteinase 9 (MMP9), N‐terminal pro‐B‐type natriuretic peptide (NT‐proBNP), suppression of tumorigenicity 2 (ST2), soluble interleukin‐6 (SIL‐6) and atrial natriuretic peptide (ANP). Characteristic molecular barcodes derived from the corresponding average fingerprint spectra with spectral differentiation between EV subgroups of h) raw blood plasma, i) mid EVs, j) large EVs and k) small EVs.

Advanced ANN SkiNET analysis on the EVs collected from blood plasma from CVD patients revealed clearly separated clusters, recapitulating the fine spectral differences of the molecular fingerprinting spectra, effectively discriminating the three EV subgroups from raw blood plasma (**Figure** **3**c). In SkiNET, inspired by the visual cortex in the brain, SOMs are trained for the neighboring neurons to activate according to similar inputs, that is, Raman spectra. Each neuron has a weight vector with length equal to the number of variables in a spectrum. Through subjecting the network to training samples over a number of iterations, the weights are gradually adjusted to become similar to the input data, to facilitate each neuron's only activation for a unique spectral signature. This yields a projection of hyperspectral data into 2D space, displayed as visible clustering according to the class, for example, sample, disease type or severity. SKiNET inherently comprised of SOMDI, which appends a set of label vectors to each neuron and allows to identify the most prominent features causing the activation of a particular neuron to a class label. The higher SOMDI intensity, the greater is the importance of particular inverse centimeters along the axis of a spectrum. Subsequently, a supervised learning step is introduced to optimize the network and the class label associated with each neuron rapidly identifies new data presented to SOM, yielding simultaneously rich‐information and high‐classification specificity, even for low laser powers and short acquisition times, representative of the real‐world diagnostic settings. Neurons (hexagons) are coloured according to the modal class they activate from the training set of Raman spectra and those with no majority class or no activation from the training data appear as white in SOMs. Combination of RS with SKiNET to investigate the disease state of a patient, identifies which spectral features, equivalent to the biochemical changes, are responsible for clustering and rapidly discriminates disease, for example, CVD, from a pool of healthy control groups.

Subsequently, we carried out EVs profiling to obtain molecular‐level information including the composition of EVs as well as identifying biomolecules specific to EVs, for example, lipids, proteins and nucleic acids. When CVDs occur clinically, the metabolites and protein contents in the blood plasma and saliva increase and the spectroscopic peaks indicative of these change in correlation within the detected spectra. The intensities of the primary peaks of interest at 960, 1003, 1136 and 1657cm^−1^ could subsequently, be used not only for the differentiation of the CVD but also, to monitor the progress post cardiac or inflammatory event since the ratio of characteristic peaks changes depending on the pathological status (Figure 3a), visible from the obtained stenting. We used the hybrid AIMSPec‐LoC with AI to assess the patients’ spectroscopically profiled data and analyze the ability to differentiate CVD from circulating EVs in blood plasma, whilst identifying the underpinning biochemical changes associated with the disease (Figure 3d–f). From the SOM/SOMDI we determined the intrinsic classification accuracy of 97.1% for the large EVs, 98.1% for the mid EVs and 100% for the small EVs, clearly discriminating between CVD and healthy cohorts (**Table** **2**).

**Table 2:** CVD Disease Differentiation via EVs. Classification performance and accuracy of healthy and CVD patient in blood plasma compared to healthy controls including sensitivity, specificity, positive predictive values (PPV) and negative predictive values (NPV).

Differentiation	Sensitivity [%]	Specificity [%]	PPV [%]	NPV [%]	Accuracy [%]
**Healthy vs CVD Raw plasma**	95.2 ± 0.7	100 ± 0.4	100 ± 0.3	95 ± 0.7	97.5 ± 0.2
**Healthy vs CVD ABs**	97.4 ± 1.1	96.8 ± 0.5	96.8 ± 0.9	97.5 ± 0.9	97.1 ± 0.5
**Healthy vs CVD MVs**	97.5 ± 0.9	98.7 ± 0.6	98.7 ± 01.2	97.5 ± 0.9	98.1 ± 0.3
**Healthy vs CVD Exos**	100	100	100	100	100

To evaluate the discrimination of the differing EV populations within blood plasma samples of patients post cardiac event, the acquired spectra were barcoded via the dominant peaks of the highest intensity and spectral differences, comparing between the CVD and control cohorts (Figure 2 j–m). Further to the average spectra, the most prominent SOMDI extracted (Figure 3c) spectral features, responsible for the clustering observed in SOM (Figure 3c, inset) include the 759, 785, 882, 960, 1003, 1124, 1131, 1307, 1336, 1440, 1600, and 1657cm^−1^. With the detailed biochemical attributions of these peaks, summarized in **Table** **3**, the most prominent characteristic changes are attributed to the nucleic acids (720–820cm^−1^), phenylalanine (1003cm^−1^), glycine backbone and proline side chain (1341–1348cm^−1^) and lipid and protein markers of the CH and CH_2_ groups (1440cm^−1^), whereas proteins, lipids and amino acids dominating the overall spectra, in correspondence with the literature on the varying cargo within the EV subgroups.^[^ ^19^, ^42^, ^71^, ^72^, ^73^ ^]^

**Table 3:** Raman spectroscopy detected assignments of the dominant, characteristic spectral peaks.^[^ ^19^, ^71^, ^72^ ^]^

Peak [cm^−1^]	Assignment
759	Tryptophan ‐ring breathing vibration
720–820	Nucleic Acids
830–853	Tyrosine doublet (protein)
875, 882	Phosphatidylcholine
920	Phosphate deformation and bending
960	Polysaccharide Structure
1003	Phenylalanine, carotenoids
1045	Proline
1124	Lipids
1131	C‐N stretching of proteins
1125	Myoglobin (haem core), phospholipids, proteins
1307	Phospholipids, lipids, adenine, myoglobin (haem core)
1341–1348	Glycine backbone and proline side chain
1368	Tryptophan, guanine, thymine, myoglobin (haem core)
1440	Lipid and protein
1450	CH_2_‐deformation
1472	CH_2_ bending of lipids and proteins
1550	Amide II/Protein
1553	Tryptophan
1656	Lipid/Amide I
1657	C = C stretching in lipids
1658	(v(C = O)), Amide I and lipids

Further key differences provide insights into the separation of detected subgroups as well as the raw blood plasma of CVD patients (Figures 3c/ S5 and S6, Supporting Information) with a major intensity difference detected at 960cm^−1^ (polysaccharide structure), 1003cm^−1^ (phenylalanine), 1341 m^−1^ (glycine backbone), 1440cm^−1^ (lipid and protein) and 1656cm^−1^ (Amide I/lipid). For the filtered EVs, the final fraction containing the small EVs yields a significantly lower peak intensity at 1440cm^−1^ yet an increased intensity at 1341cm^−1^ (p***<0.0001), attributed to glycine backbone, while each of the three EV subgroups exhibits less intense lipid and protein peaks, that is, 1440 and 1656 cm^−1,^ than raw blood plasma. A significant decrease is also measured in protein concentration at each subsequent filtration step in the LoC, pertinent to raw blood plasma, large, mid and small EVs (Figure 3b). Blood plasma is known to contain a wide range of proteins including albumins, clotting factors, enzymes and hormones as well as various other proteins involved in immune response, transport, and regulation.^[^ ^74^ ^]^ On the other hand, EVs protein content varies depending on the cell type and conditions and these are known to carry a subset of proteins derived from their cells of origin with their cargo involved in intercellular communication and cell‐to‐cell signalling. While EVs can contain a diverse range of proteins, they are typically present at much lower concentrations compared to blood plasma^[^ ^75^ ^]^ (Figure 3b). Amongst the three EV subgroups, small EVs exhibit the least strong peak attributed to proteins whilst the large EVs, the most intense. Large EVs, such as apoptotic bodies are released during programmed cell death, that is, apoptosis, and are larger in size compared to the other EV subgroups and contain a diverse range of proteins derived from the dying cell.^[^ ^25^, ^76^ ^]^ Small EVs, such as exosomes, being considerably smaller and formed by the inward budding of multivesicular bodies, also contain further proteins including, membrane and cytosolic proteins. However, they generally have lower protein levels compared to larger apoptotic bodies due to their size and the selective packaging of cargo during their biogenesis, corresponding to the lesser measured protein content.^[^ ^76^, ^77^, ^78^ ^]^

Determining the total protein concentration of a specific subgroup is further useful in assessing the separation efficiency of the LoC designed for EVs isolation. A higher protein concentration in the retained fraction indicates better capturing and enrichment of the subgroup and thus, a higher efficiency of the LoC device in isolating a specific EV population. Conversely, if the protein concentration is similar in both fractions, it indicates limitations in the separation efficiency for the targeted subgroup. In the AIMSPec‐LoC, as the vesicles passed through the platform, the overall protein concentration decreases, indicating effective size‐based separation. To further address the challenge associated with EV isolation and separation due to contaminations, the LoC was designed to allow contaminating proteins to flow through and since the majority are smaller than EVs, these contaminants were subsequently trapped by the 40 nm filter. This results in successful separation of contaminants since the overall protein concentration has decreased during the blood plasma passing through each filtration step (Figure 3b), in agreement with the 92% separation efficiency calculated via a combination of fluorescence microscopy and NTA (Figure 2i).

A further statistically significant difference is found for the dominant bands at 1124, 1440, and 1656cm^−1^ (p***<0.0001) associated with lipids at decreased intensities in EVs compared to raw blood plasma, with small EVs exhibiting the lowest levels (Figure 3g,h). Blood plasma contains a variety of lipids, including triglycerides, cholesterol, phospholipids and fatty acids, which are essential for physiological functions including energy storage, membrane structure and signalling.^[^ ^74^ ^]^ In contrast, when EVs are isolated, the specific method employed to separate the vesicles from the bulk of the blood plasma typically removes lipids which are not associated with EVs, resulting in a relatively lower lipid content in isolated EVs compared to the raw plasma.^[^ ^31^ ^]^ Bands at 882cm^−1^ (phosphatidylcholine) and 1553cm^−1^ (tryptophan) exhibited significantly increased intensities in EV subgroups relative to the raw blood plasma. Combined with the changes observed in protein and lipid peaks (i.e., 1124, 1440, 1656cm^−1^), these bands could be employed as spectral markers for the EV characterisation, which would also aid in the development of further studies to assess the clinical utility of the developed technology.

Disease indicative EV spectral differences are found to vary significantly with the representative Raman spectra of healthy and CVD derived EVs for each subgroup exhibiting major intensity changes (Figure 3d–f). Both, the mid EVs and the small EVs, exhibited significant intensity increases (≈17–68%) (p**<0.001) in CVD group at bands of 752cm^−1^ (nucleic acids), 851cm^−1^ (tyrosine), 960cm^−1^ (polysaccharide structure), 1144cm^−1^ (lipids), 1440cm^−1^ (lipids and proteins) and 1656cm^−1^ (Amide I/lipids) with an additional increase at 1003cm^−1^ for small EVs. The increased intensities of nucleic acids (Figure 3e,f) can be attributed to the EVs’ representing an important intracellular communication mechanism through the containment and transportation of bioactive molecules such as, proteins and micro‐ribonucleic acids (miRs) to target cells.^[^ ^14^, ^79^, ^80^ ^]^ EVs therefore, pass on favourable, neutral or harmful effects on recipient cells, which include modulating gene expression and affecting molecular pathways. In the case of cardiovascular diseases, EVs can be released by cardiovascular‐related cells such as, platelets, monocytes, leucocytes and cardiomyocytes^[^ ^8^ ^]^ and are capable of inducing pathological changes associated with CVDs as the composition of proteins and miRs transferred by EVs, which maintain cardiovascular balance, can be altered thus, giving rise to the development of CVDs.^[^ ^81^ ^]^ Patients with CVDs often exhibit increased levels of nucleic acids circulating in their blood, resulting in packaging of these within the EVs and subsequent release into the blood stream.^[^ ^82^ ^]^

The detected increase in intensity of the amino acid tyrosine in CVD‐derived EVs would indicate an associated increased risk for CVD. Tyrosine is the precursor of catecholamines, which cause high blood pressure when found at increased levels in the body.^[^ ^83^ ^]^ Previous studies have shown that tyrosine causes tachycardia and hypertension in small doses, whereas in larger doses the opposite was observed, that is, bradycardia and hypotension.^[^ ^84^ ^]^ Closely associated with the effect of elevated tyrosine levels in CVDs, is the increased concentration of phenylalanine. In two of the investigated EV subgroups, we have found both peaks at 1003cm^−1^ associated with phenylalanine as well as the 851cm^−1^ associated with tyrosine are increased in CVD patients. Inflammation and immunological activation are directly linked to the onset and progression of CVDs, with indications that they furthermore hamper the conversion of phenylalanine to tyrosine in individuals with clinical disorders. Intrinsically, increased phenylalanine concentrations and a higher phenylalanine‐to‐tyrosine ratio have been linked to immunological markers^[^ ^85^ ^]^ thus, suggesting a potential spectral biomarker of CVD. The observed differences in intensity of each EV subgroup compared to raw blood plasma for both tyrosine and phenylalanine indicate the different biochemical composition of EVs isolated via the LoC. Whilst amino acid levels in plasma have been used for early detection and diagnosis of diseases such as cervical cancer, pancreatic cancer and type 2 diabetes, differing amino acid levels in EVs have only been reported in small EVs analyzed with high performance liquid chromatography with fluorescence,^[^ ^86^ ^]^ where distinct differences were observed between small EVs and native serum amino acid compositions. Of a particular interest, in the study by Onozata *et* al. histidine, arginine, glutamic acid, cysteine, lysine and tyrosine were found to be significantly increased (p*<0.05) in exosomes, indicating that certain amino acids are enriched in exosomes. We have similarly identified the most significant increase in amino acids (tyrosine and phenylalanine) in the fraction associated with exosomes, in agreement with the study by Onozato *et al.* ^[^ ^86^ ^]^ In addition, the 1553cm^−1^ peak associated with tryptophan has exhibited a significantly decreased intensity (p*<0.05) in CVD blood plasma in each of the three EV subgroups. Tryptophan breakdown into downstream metabolites is accelerated at the onset of CVD via interferon‐*γ*‐mediated inflammation.^[^ ^87^ ^]^ Yu *et* al. determined that a lowered risk of CVD was strongly correlated with an increase in plasma tryptophan content, in line with the findings presented here, whereby tryptophan is inversely associated with the incidence of CVD.^[^ ^88^ ^]^

To further categorize and determine disease‐specific molecular markers within the EVs, RS was used to profile and classify a panel of CVD‐indicative biomarkers. The identified panel of CVD biomarkers including apolipoprotein B (ApoB), lipoprotein A (LpA), proprotein convertase subtilisin/kexin type 9 (PSCK9), C‐reactive protein (CRP), d‐dimer, interleukin‐9 (IL‐9), myeloperoxidase (MPO), tumour necrosis factor‐alpha (TNF‐α), copeptin, galectin‐3, growth and differentiation factor‐15 (GDF‐15), matrix metalloproteinase 9 (MMP9), N‐terminal pro‐B‐type natriuretic peptide (NT‐proBNP), suppression of tumorigenicity 2 (ST2), soluble interleukin‐6 (SIL‐6) and atrial natriuretic peptide (ANP) were chosen based on their specificity for CVD and known physiological response in patients. The acquired average Raman spectral fingerprints from the panel of these biomarkers are shown in Figure 3i. The spectral fingerprints of healthy compared to the CVDs with ApoB, LpA, NT‐ProBNP, PCSK9 and IL‐9 exhibit a statistically significant difference (p*<0.05) with an increased level of each marker in CVD‐derived EVs, which is most likely due to atherosclerosis affecting the coronary arteries, primarily influenced by cholesterol and specifically, low‐density lipoprotein cholesterol (LDL‐C).

This is further underscored by the success of LDL‐C lowering medications, such as statins as well as Mendelian randomization studies, which clearly demonstrated the causative role of LDL‐C's in CVD.^[^ ^89^, ^90^ ^]^ For instance, Leander *et* al., identified the significant role of PCSK9 in the regulation of LDL‐C and thus, demonstrated the potential of soluble PCSK9 to act as a novel indicator of CVD risk.^[^ ^91^ ^]^ Further, the LpA, ApoB and PCSK9 demonstrated increased intensities at 1315cm^−1^ as well as between 1200–1340cm^−1^ are attributed to lipids and the identified upregulation of Amide I (1600–1655cm^−1^) in both NT‐ProBNP (≈18%), IL‐9 (≈14%) and LpA (≈32%), suggests the significant role of lipids and cytokines in the onset of CVD and demonstrates their potential as significant indicators of cardiovascular disease (Tables S1–S4, Supporting Information).

Further, via the application of SKiNET in the data classification, spectral barcodes of healthy and diseased EV subgroups for CVD have been derived from the corresponding average fingerprint spectra selected from the bands of the highest intensity and significance (Figure 3j–m) with SOMDI extracted features from SOMs, highlighting the most influential Raman peaks for each class. This establishes the multiplex barcoding from a complex biological matrix based on their distinct spectral signatures combined with the computational SKiNET algorithm for rapid classification, via the key‐features from the spectral analysis visually represented in the coloured Raman maps, providing a selective and sensitive method for detection of CVD. In addition to blood plasma EVs offering instrumental roles in disease diagnosis, EVs isolated from saliva hold further important diagnostic potential, particularly due to its non‐invasive and readily accessible characteristics. The versatile nature of the developed AIMSPec‐LoC easily adaptable to different biofluids, opens a path in the successful diagnosis, and monitoring of further diseases.

**Figure 4:**
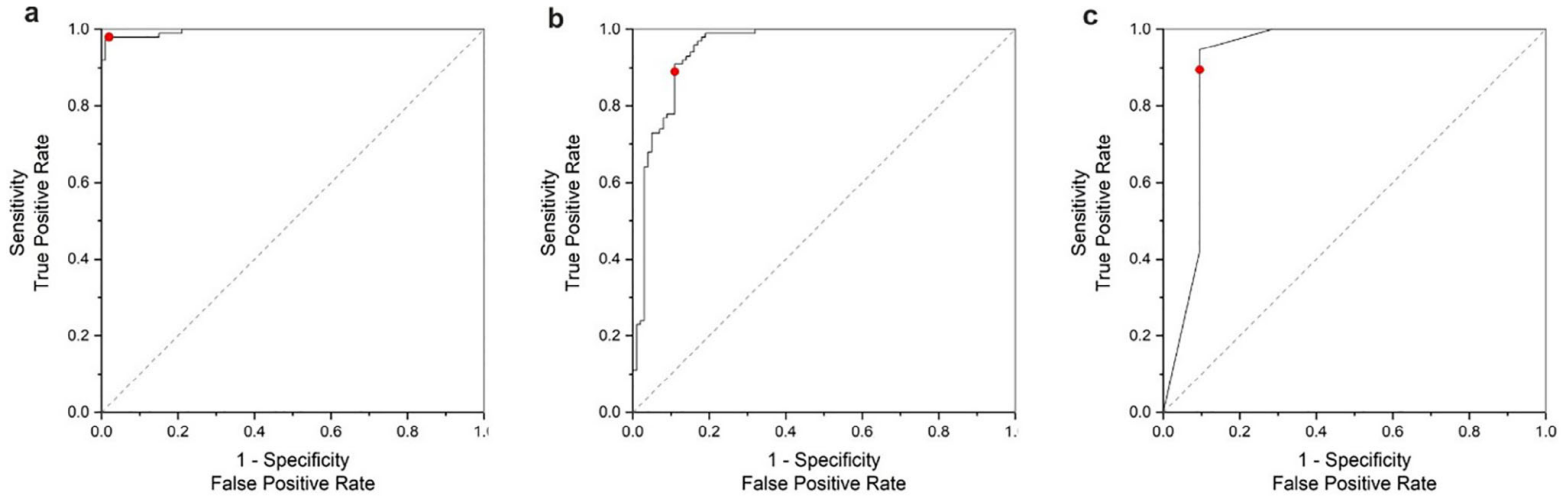
ROC Plots Representing the Sensitivity versus 1‐Specificity. ROC plots derived from post probability assignment applied to determine the success for classifying the healthy and CVD‐derived blood plasma for a) small EVs, b) mid EVs and c) large EVs. The AUC indicates that the change in the protein and lipid levels of plasma and saliva‐derived EVs are valuable markers to discriminate healthy from the CVD‐indicative EVs with the CVD indicative EVs showing an excellent performance with AUC of 0.99 (95% CI 0.99–1.0), 0.95 (95% CI 0.94–0.96) and 0.92 (95% CI 0.81–1.03) for small EVs, mid EVs, and large EVs, respectively.

We used receiver operating characteristic (ROC) curves to assess the differentiation of healthy and CVD blood plasma as well as healthy and IBD saliva for each of the EV subgroups by calculating the area under the curve (AUC), plotting the true positive versus the false negative rates (**Figure** **4**). From the ROC curves we determined the intrinsic classification accuracy in CVD‐derived blood plasma of 99% for small EVs (Figure 4a), 95% for mid EVs (Figure 4b), 92% for large EVs (Figure 4c), clearly discriminating between healthy and diseased groups, which is crucial for classifying CVD. To note, the ROC's steep ascent with the consistent trajectory toward the upper‐left corner, indicating a minimal rate of false positives while maximizing true positive predictions of the classification. This is critical when assessing diagnostic or predictive models, due to the indication of the ability to accurately detect relevant disease cases and states. The calculated AUCs overall range from 0.92 to 0.99 with the higher values demonstrated for the exosome EV subgroup in both plasma and saliva, in‐line with SOM classification accuracy (Figure 3c) with classification accuracies of >96%. These AUC values underscore the robustness of our combined AIMSPec‐LoC with intrinsic AI classification, exhibiting accurate discriminatory capabilities, particularly valuable in applications of diagnostics, where precision and specificity are of a paramount importance. Through spectral barcoding of the data sets, each serves as a spectral fingerprint, allowing for the identification and characterisation of individual biochemicals without the need for prior knowledge or reference samples. It also allows for the differentiation of closely related species with similar Raman bands as well as any changes within the barcode, which may be due to the disease progression or drug interactions, cementing the AIMSPec‐LoC suitability to non‐invasively detect and monitor the diseased state of patients.

Many previous studies have aimed to characterize EVs by sized and origin, using different methodologies including, differential centrifugation, density gradient ultracentrifugation, NTA, DLS, flow cytometry, and mass spectrometry‐based proteomics and lipidomics.^[^ ^92^, ^93^, ^94^, ^95^, ^96^, ^97^ ^]^ Several methods have successfully confirmed the heterogeneity of EV populations, with some indicating size‐based functional distinctions. For example, Kowal *et* al. identified distinct protein cargoes between small and large EVs isolated via centrifugation.^[^ ^98^ ^]^ They identified MHC I and MHC II in all EVs, GP96 and possibly other ER‐associated proteins were mainly present in large EVs, actinin‐4 and mitofilin are present in both large and mid‐EVs but absent in small EVs and syntenin‐1, TSG101, ADAM10, and EHD4 are only present in small EVs with syntenin‐1 and TSG101 specific to the tetraspanin‐enriched small EVs representing exosomes. Similarly, Lischnig et al. also identified varying proteins enriched in either small or large EVs.^[^ ^99^ ^]^ The authors also identified tetraspanins, ADAMs and ESCRT proteins were enriched in small EVs whereas ribosomal, mitochondrial, and cytokinesis proteins are enriched in large EVs thus, suggesting protein markers for large and small EVs. Théry et al. have also suggested that vesicles may differ in tetraspanin expression or lipid content.^[^ ^100^ ^]^ However, the majority of EV studies rely on bulk isolation and characterisation methods which lack the resolution to distinguish biochemical differences at the level of individual EV subpopulations, and often cannot assess multiple molecular modalities simultaneously (e.g., proteins, lipids and amino acids). Additionally, techniques such as flow cytometry, while useful for surface marker detection, are limited by the need for labelling, poor sensitivity for small EVs (<200 nm) and may present bias toward pre‐selected markers, therefore uncharacterized or minor vesicle subtypes may be excluded.^[^ ^101^ ^]^

In contrast, the study presented here used *label‐free* RS to assess the intrinsic molecular composition of small, mid, and large EVs, revealing significant biochemical distinctions among these subgroups in terms of protein, lipid, and amino acid spectral features. Unlike previous approaches that often primarily focus on a specific size or surface marker, RS enabled the analysis of the overall molecular “fingerprint” of each EV population in a minimally processed state. Importantly, we observed that not only do small, mid, and large EVs differ in their Raman spectral signatures, which indicates compositional heterogeneity, but each of these subgroups also exhibited distinct Raman intensity changes between healthy and CVD plasma samples. This multiplexed layered separation – both in size and diseased state – goes beyond most of what has previously been reported where often bulk EV populations from diseased and healthy donors are evaluated without resolving within‐population heterogeneity. Moreover, proteomic studies such as those by Goetzl et al. and Freeman et al. have identified disease‐associated changes in EV protein cargo, however such analyses typically require extensive processing, larger sample volumes, and do not correlate findings with EV size.^[^ ^102^, ^103^ ^]^ Here, the Raman analysis captured biochemical differences related to CVD across size‐resolved EV populations without the need for prior labelling, fractionation, or enrichment, and the observed spectral differences corresponded with expected disease‐associated changes, such as increases lipid‐associated peaks and altered amino acid intensities, which have previously been reported in CVD plasma profiling. Furthermore, the incorporation of ML into the spectral analysis presented here allowed the classification of EV subtypes and disease states with high accuracy. Traditional characterisation techniques such as NTA and DLS which focus on purely physical parameters lack such molecular insight. By combining high‐dimensional spectral data with ML, nuanced patterns that provide both mechanistic insights into EV biology and potential clinical utility in diagnostics and monitoring may be uncovered.

While conventional approaches such as flow cytometry and proteomics have demonstrated utility in EV characterisation, they often exhibit lower sensitivity for early‐stage disease detection and require complex, multi‐step protocols. In contrast, our AIMSPec‐LoC platform achieved diagnostic accuracies of >97% across all EV subtypes, outperforming many existing tools in terms of both speed and resolution, especially for small EVs which are poorly detected by flow cytometry (<200 nm). Furthermore, the integration of Raman spectroscopy with lab‐on‐a‐chip technology offers a compact, scalable solution suitable for real‐time, point‐of‐care diagnostics, whereas most conventional methods lack portability and are limited to specialized laboratory settings.

## 3 Conclusions

Cardiovascular diseases are often diagnosed only after acute, life‐threatening events such as myocardial infarction or stroke. This underscores an urgent need for technologies capable of detecting the subtle biochemical changes that precede these events, thereby enabling early diagnosis and intervention. Our study addresses this critical gap by presenting a novel platform, AIMSPec‐LoC, for the simultaneous isolation and analysis of EVs, which are increasingly recognized as robust biomarkers for early‐stage CVD. The AIMSPec‐LoC achieves simultaneous separation and analysis of EV subtypes, including large, mid and small EVs without requiring extensive sample preprocessing. This innovation preserves EV integrity and cargo, enabling multiplexed, real‐time molecular profiling. By delivering a comprehensive characterization of EV subpopulations and their biochemical signatures in a single, streamlined workflow, the platform provides substantial new insights into the pathophysiology of CVD. Importantly, the AIMSPec‐LoC demonstrated outstanding diagnostic performance, achieving >96% sensitivity and specificity in the detection of disease‐specific EV markers across multiple biofluids, underscoring its clinical relevance and translational potential. The platform's design allows scalability to accommodate larger sample volumes, enhancing its applicability for diverse diagnostic needs. Furthermore, integration with advanced handheld Raman and machine learning algorithms, such as SKiNET, enables the generation of high‐dimensional barcoded datasets, facilitating multiplexed molecular profiling and robust classification of EV‐associated biomarkers. These capabilities not only accelerate the discovery of disease mechanisms but also offer a pathway toward real‐time monitoring of disease progression and therapeutic response.

The AIMSPec‐LoC represents a significant technological advance in non‐invasive diagnostics, addressing critical limitations of conventional approaches such as tissue biopsies by reducing patient discomfort and improving safety. Its ability to quantitatively assess EV biomarkers at the earliest stages of CVD holds promise for advancing clinical decision‐making, enabling earlier therapeutic interventions and improving patient outcomes.

This work establishes a foundational framework for the future development of in‐vivo clinical measurements using portable AIMSPec‐LoC device. Beyond CVDs, the versatility of this platform positions it as a valuable tool for timely translational and basic science related to the complex interactions between organs in heart failure, cardiometabolic diseases and multimorbidities including for instance the gut‐heart axis, neuro‐cardiac interactions, immune responses and the impact of aging on organ crosstalk. The adaptability of the AIMSPec‐LoC to diverse biomarkers offers an untapped potential for improving diagnostic accuracy and patient outcomes across a broad spectrum of heart conditions. By enabling timely interventions, this technology has the potential to significantly reduce morbidity and mortality associated with devastating CVDs, ultimately transforming global healthcare management.

## 4 Experimental Section

### Clinical Samples

Study participants were recruited through the Inflammatory Bowel disease and Cardiovascular Research Centres at Queen Elizabeth Hospital of Birmingham (UK) as part of the CASCADE and TRAFIC studies) (Ethics Ref.19/SW/0010). Written informed consents were received from participants or valid proxy (family or a professional not directly involved in the study) prior to inclusion in the study. The study was approved by the National Research Ethics Service (Research Ethics Committee reference RRK6508 and ERN_22_0290). Both TRAFIC and CASCADE studies comply with the guidelines of the Declaration of Helsinki. The samples were obtained from a standard, widely accepted classification of the CVD based on clinical criteria, that is, full blood panels as well as supported by in‐hospital scans including electrocardiograms for CVD diagnosis. Patients were categorized into HV, CVD. All patients were gender and age matched to HVs. Patient demographics are shown in Table 1. Blood samples from patients in each category were obtained at QEHB. Once collected, they were centrifuged initially at 2000×g for 20 minutes and then, via a further centrifugation cycle of 13 000×g for 2 minutes. Extracted blood plasma was stored at −80 °C until analysis. All samples were processed within 2 h of venepuncture.

### Blood Plasma Collection and Preparation

Blood samples were collected from Queen Elizabeth Hospital Birmingham and University of Birmingham, UK from 40 participants. Among them, 20 were clinically diagnosed with CVD confirmed via current clinical practices and the remaining 20 participants were healthy with no known health issues or conditions. Blood plasma was separated from whole blood using a 2‐step centrifugation process; initial centrifuge at 2000×g for 20 minutes, followed by removal of supernatant and further centrifuge at 13 000 × g for 2minutes. Plasma was then extracted and stored at −80 °C. During the recruitment process, written informed consent was obtained from each participant.

### Nanoporous Membrane Chip

All layers were fabricated using 3D resin printed micro moulds of high temperature resolution resistance resin (Siraya Tech, USA). Moulds were printed using a monochrome LCD/MSLA resin printer equipped with a 405 nm light source (Elegoo Mars3) and subsequently, thoroughly washed in isopropyl for 20minutes, annealed at 120 °C and finally, cured under an ultraviolet lamp (Elegoo, China) for 20minutes to prevent mould/PDMS interaction. PDMS (Dow corning, USA) was mixed in a ratio of 5:1 with curing agent Sylgard 184 (Dow Corning, USA). Access holes of 6 mm diameter were devised for the inlet and outlet chambers, respectively. Sterlitech polyethersulfone (PES) membrane filters of 25 mm diameter with varying pore sizes (5000, 500, 200, 100, and 40 nm) were placed between the PDMS layers. PDMS layers were bonded through a combination of plasma for 10minutes followed by hard baking at 120 °C for 30minutes.

### Lab‐on‐a‐Chip Device Design and Fabrication

The designed microfluidic chip consisted of 5 layers of PDMS adhered to a glass slide. The top layer contained a well for the sample inlet, at the base of the well and between the top and middle layers a hydrophilic polyethersulfone nanoporous membrane with a pore size of 5000 µm to filter out any cellular debris/food particles. Saliva dripped through the filter into the inlet of the next PDMS layer, moving down the microfluidic channel and into the sample collection area containing the PBS buffer. It subsequently, passed through a further filter membrane (500 nm) sandwiched between the two PDMS layers, where EVs of 5000–500 nm (large EVs) were trapped and collected. Saliva continued to drip through a further three layers with pore sizes of 200, 100, and 40 nm to collect and trap EVs in the size ranges of 500–200 (mid EVs), 200–100 (small EVs), 40–100 nm (small EVs). 40 nm filter was used to trap proteins. Capillary channels were used to aid flow throughout the filtering process. Prior to sample injection, 1 mL of PBS was injected into the inlet to pre‐wet the filter membranes. Sample outlet was filled with 100 µL of PBS to aid capillary flow throughout the system. After 10minutes, inlet PBS was replaced with 100 µL of sample. Separation was carried out at room temperature.

### Nanoparticle Tracking Analysis

NTA NanoSight NS300 (Malvern, UK) was used to measure the size distribution and concentration of the collected EVs. Blood plasma samples were diluted with pre‐filtered PBS in a 1:5 ratio with saliva diluted 1:10 in line with recommended particle concentration per frame (20–100 particles/frame). For each measurement five 60s videos were captured. After capture, NanoSight software NTA3.1 was used to analyze video segments for each EV measurement with a detection threshold of 6.

### Dynamic Light Scattering

DLS measurements were performed using the Malvern Panalytical Zetasizer HPPS equipped with a 633 nm He‐Ne laser. Low volume quartz cuvettes were used for analysis of 15 µL of samples. All measurements were carried out at a fixed position with an automatic attenuator and at a controlled temperature of 25 °C. Five measurements were averaged for each EV sample.

### Fluorescence Microscopy

EVs were analyzed using Carl‐Zeiss LSM780 microscope equipped with excitation wavelengths of 405, 488, and 514 nm to visualize coloured nanoparticles with ×50/×100 objective lens. All images were processed using ImageJ software.

### Scanning Electron Microscopy

EVs were analyzed on the filter membrane, in PBS and in fixed form. EVs were fixed on silicon wafers using 2.5% glutaraldehyde prepared in PBS for 30minutes. The sample was then sequentially dehydrated in an ascending sequence of ethanol. Samples were dried at room temperature prior to SEM observation. Several randomly selected frames from each sample were captured for morphological assessment and scanning electron micrographs were acquired using a thermally assisted Field Emission Scanning Electron Microscope (FESEM, LEO VP 1530/FEI Helios) with a lateral resolution of 2–5 nm. A LEO ULTRA 55SEM instrument including a Schottky emitter (ZrO/W cathode) was also used for imaging the samples with a typical acceleration voltage of 2–5 kV.

### Raman Spectroscopy

Raman spectroscopy was performed directly on chip at each filter membrane. Raman spectra were acquired using a Renishaw InVia Qontor confocal Raman microscope equipped with a microscope Leica DMLM (Renishaw PLC, UK). 785 nm excitation laser was used in the analysis and laser light was focused using ×50 objective. Output power at the sample was 5 mW. All Raman spectra were acquired in the main fingerprint region of 700–1700cm^−1^. Map scans were obtained over a 50 × 50µm^2^ areas for each sample using a 5 µm step size between map points, 10 accumulations of 1 s acquisition per spectra. In summary, 1 map with 100 spectra were collected per sample for both saliva and plasma and used for data processing and analysis. All Raman data was acquired using WiRE 5.1 (Renishaw PLC, UK) also applied for the polynomial background subtraction and removal of the cosmic rays. Spectra were normalized using the standard normal variate (SNV) using Python (v.3.7). Signal‐to‐noise ratio was improved using 10 averaged spectra at each map location.

### SKiNET Classification

Multivariate Analysis was performed using the SKiNET, alongside the Raman Toolkit web interface to build the SOM models. Data sets were grouped and split into test and training data (20:80). Tenfold cross validation was performed on the training data with optimisation of grid‐size, initial learning rate and number of training steps. The end model used to classify the test data consisted of a 10 × 10 grid of neurons, 46 080 training steps (4 epochs of the data) and an initial learning rate of 0.1. Python was used to complete multi‐chemical barcoding of data. Savitzky‐Golay filter was applied to calculate the second derivative of each spectrum. Smoothing window was set to 21 with a polynomial order of 2. Maximum peak heights with absolute values over 40% were assigned a value of 1 with values below 40% assigned a value of 0. Values were overlaid on the averaged spectra with reference to the main peaks of interest identified, thus creating the characteristic barcode.

### Statistical Analysis

Spectral data collected was analyzed using IBM SPSS Statistics (IBM Corp., USA). Descriptive statistics, including means, standard deviation and range were calculated to summarize demographic characteristics of the study participants. Statistical significance of the difference between two sets of data was assessed using the Wilcoxon rank sum/Mann‐Whitney U test. The significance level for all statistical tests was set at 0.05. A *p*‐value <0.05 was considered statistically significant. Comparisons across groups at each time and within groups over time were performed by the analysis of variance and Tukey's post hoc test on transformed data.

### ROC Curves and Box Plots

ROC curves were generated from patient profiling data for different cut‐off points using non‐parametric Mann‐Whitney U and Kruskal Wallis tests, run using SPSS statistics software. Each point in the ROC curve represented sensitivity/specificity pair corresponding to a particular decision threshold and the diagnostic values of sensitivity, specificity, and accuracy were calculated using standard equations. A test with perfect discrimination (no overlap in the two distributions) had an ROC curve that passed through the upper left corner (100% sensitivity, 100% specificity). The closer the ROC curve was to the upper left corner, the higher was the overall accuracy of the test. Box plots were generated using the Vertex42 LLC software where each of the series is an x‐y chart used to represent the quartiles and allows the data to include negative values. The median is represented with an “x” marker and horizontal markers are used for Q_1_ and Q_3_ without requiring shifting of the data. The mean levels of patients’ group comparison to healthy volunteers control group were performed using two‐sided normal‐based 95% CIs *t*‐test. Classification sensitivity, accuracy and specificity were determined on the basis of disease detection results: Sensitivity = (TP)/(TP+FN), Specificity = (TN)/(TN+FP) and Accuracy = (TP+TN)/(TP+TN+FN+FP) with TP being “true positive,” TN “true negative,” FP and FN “false positive” and “false negative,” accordingly.

## Conflict of Interest

The authors declare no conflict of interest.

## Author Contributions

E.B.: Conceptualization, investigation, methodology, formal analysis, writing‐original draft, review and editing. J.J.S.R.: Conceptualization, investigation, methodology, writing‐original draft, review and editing. M.T.: Patient recruitment, writing‐ review and editing. P.G.O.: Conceptualization, methodology, investigation, resources, patient recruitment writing‐original draft, review and editing, funding acquisition, supervision. All authors reviewed and approved the final version of the manuscript.

## Supporting information



Supporting Information

## Data Availability

The data that support the findings of this study are available in the supplementary material of this article.
