# Supplementary material for: Advanced Multipurpose Spectroscopic Nanobio‐Device for Concurrent Lab‐on‐a‐Chip Label‐Free Separation and Detection of Extracellular Vesicles as Key‐Biomarkers for Point‐of‐Care Cardiovascular Disease Diagnostics
Source: Adv Healthc Mater. 2025 Jun 2;14(21):2500122. doi: 10.1002/adhm.202500122 (PMC12365627; doi:10.1002/adhm.202500122)
Supplement: Supplementary file 1 — Supporting Information [file ADHM-14-0-s001.docx]

**- Supporting Information -**

**Advanced Multipurpose Spectroscopic Nanobio-device for Concurrent Lab-on-a-Chip Label-free Separation and Detection of Extracellular Vesicles as Key-biomarkers for Point-of-Care**

**Cardiovascular Disease Diagnostics**

Emma Buchan^1^, Jonathan James Stanley Rickard^2^, Mark Robert Thomas^3^, Pola Goldberg Oppenheimer^1,4,*^

*^1^School of Chemical Engineering, College of Engineering and Physical Science, University of Birmingham, Birmingham, B15 2TT, UK*

*^2^Department of Physics, Cavendish Laboratory, University of Cambridge, JJ Thomson Avenue, Cambridge, CB3 0HE, UK*

*^3^Institute of Cardiovascular Sciences, College of Medical and Dental Sciences, University of Birmingham, Birmingham, B15 2TT, UK*

*^4^Healthcare Technologies Institute, Institute of Translational Medicine, Mindelsohn Way, Birmingham, B15 2TH, UK*

**S1. LoC Performance:**

**Figure S1. Time Efficiency of Loc Saliva Filtration.** Overall time taken to filter saliva through the LoC. Based on 10 sample repeats with error bars representing standard deviations.

**Figure S2. Time Efficiency of Loc Blood Plasma Filtration.** Overall time taken to filter blood plasma through the LoC. Based on 10 sample repeats with error bars representing standard deviations.


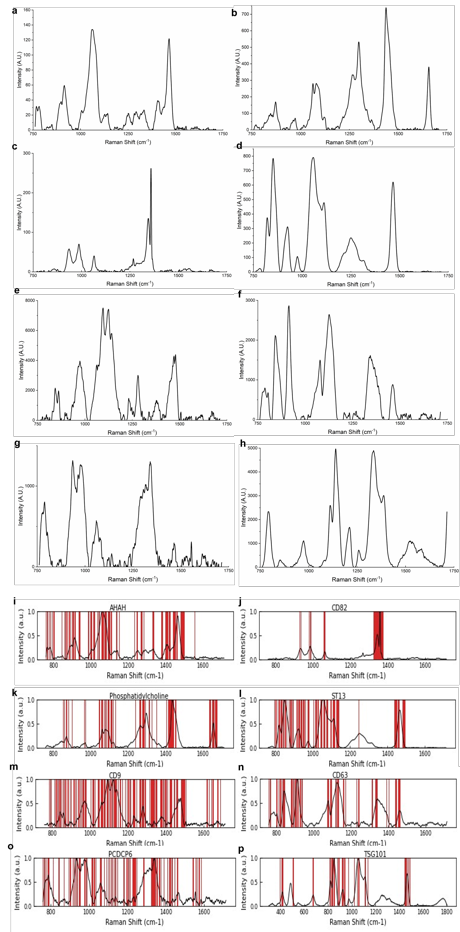
**S2. EV Indicative Biomarkers:**

**Figure S3. EV Indicative Biomarkers**. **(a)** AHA1, **(b)** phosphatidylcholine, **(c)** CD82 and **(d)** calnexin **(e)** CD9 **(f)** CD63, **(g)** PCDC6IP and **(h)** TSG101. Molecular barcoding of corresponding biomarkers including the **(i)** AHA1, **(j)** CD82 and **(k)** phosphatidylcholine, **(l)** calnexin, **(m)** CD9, **(n)** CD63, **(o)** PCDC6IP and **(p)** TSG101.

Multiple exosomal surface markers, which are routinely used in the identification of extracellular vesicles including tetraspanins CD9, CD63 and CD81, programmed cell death 6 interacting protein (PCDC6IP) as well as tumour susceptibility gene 101 protein (TSG101), phosphatidylcholine, calnexin heat shock protein 70 and heat shock protein 90, have been evaluated. Although present in both exosomes and microvesicles, CD63, CD9 and PCDC6IP have been routinely found to be enriched in exosomes relative to the microvesicles [66], [67] while calnexin abundant in larger microvesicles [68]. Phosphatidylcholine was also analysed as a key indicator of EVs with reports suggesting phosphatidylcholine comprises 46-89% of the lipid components in all exosomes [69]. Heat shock proteins, considered as intracellular proteins, are capable to shuttle between the cytoplasm and the nucleus and although typically not identified on the surface of healthy cells, have been denoted as constitutive EV proteins [70]. From the detected biomarkers in our study, statistically significant peaks of interest (*p*** < 0.01) were identified at Raman shifts of 785, 960 and 1448cm^-1^ for the exosome group, 1250-1350 and 1656 cm^-1^ for microvesicles and 1003 and 1120cm^-1^ for the apoptotic bodies.


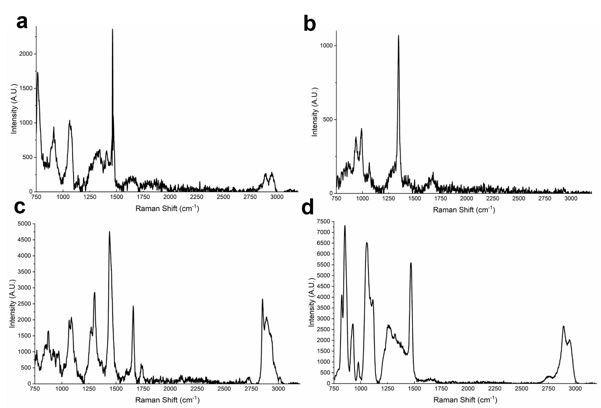


**Figure S4. Extended Raman Spectral Fingerprinting of EV-indicative Biomarkers.** Representative extended Raman spectral scans revealing the vibrational characteristics of a range of EV-indicative biomarkers including the **(a)** AHA1 - indicating prominent spectral peaks at 752, 960, 1003, 1250, 1500 and 2900 cm^-1^, the **(b)** CD82 - demonstrating noticeable shifts in vibrational modes at 972, 1003 and 1352 cm^-1^, the **(c)** phosphatidylcholine - exhibiting prominent peaks of interest at 920, 1132, 1285, 1448, 1656, 2800 and 2942 cm^-1^ and the **(d)** calnexin showing distinctive peaks at 922, 980, 1003, 1132, 1250-1350, 1500 and 2920 cm^-1^. Extended spectral coverage provides a comprehensive view of EV-indicative biomarker characteristics, allowing for further detailed analysis and interpretation of observed EV peak intensity changes.

**S3. Box And Whisker Plots of Primary Peaks Differentiating Each of The Ev Sub Populations In Blood Plasma:**

**
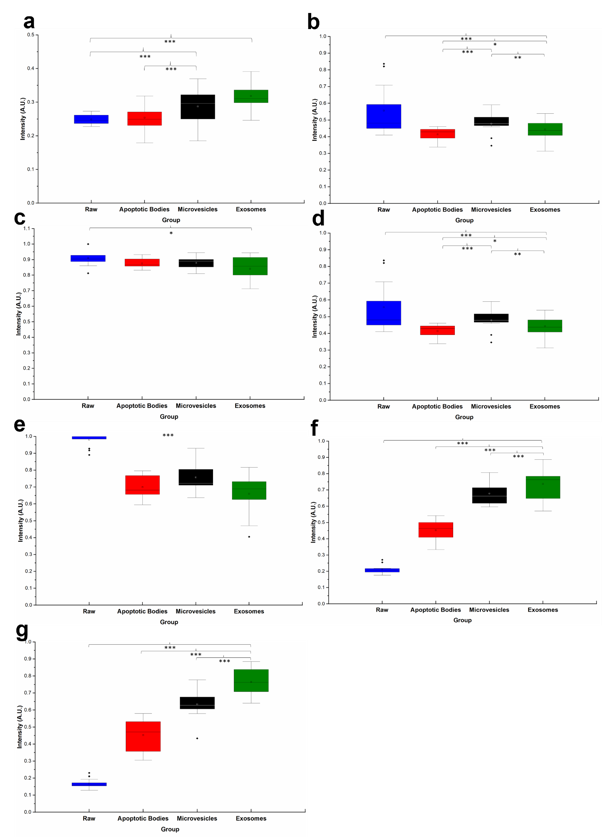
**

**Figure S5. Spectral Variability Within CVD-Indicative Blood Plasma EVs.** Box and whisker plots represent the minima, maxima, interquartile ranges, whiskers and the median in peak intensity distributions at **(a)** 851cm^-1^, **(b)** 960cm^-1^, **(c)** 1003cm^-1^, **(d)** 1320 m^-1^, **(e)** 1336cm^-1^, **(f)** 1448cm^-1^ and **(g)** 1657 cm^-1^ for healthy control CVD-indicative EV subgroups and raw CVD blood plasma. The box represents the interquartile range (IQR) with the median indicated by a line inside the box. Whiskers extend to 1.5 times the IQR, and the diamonds denote outliers beyond the whiskers. Intensity levels were significantly higher between EV subgroups and raw plasma (*p* <0.05, ***p* <0.01 and ****p*<0.0001, student’s *t*-test).


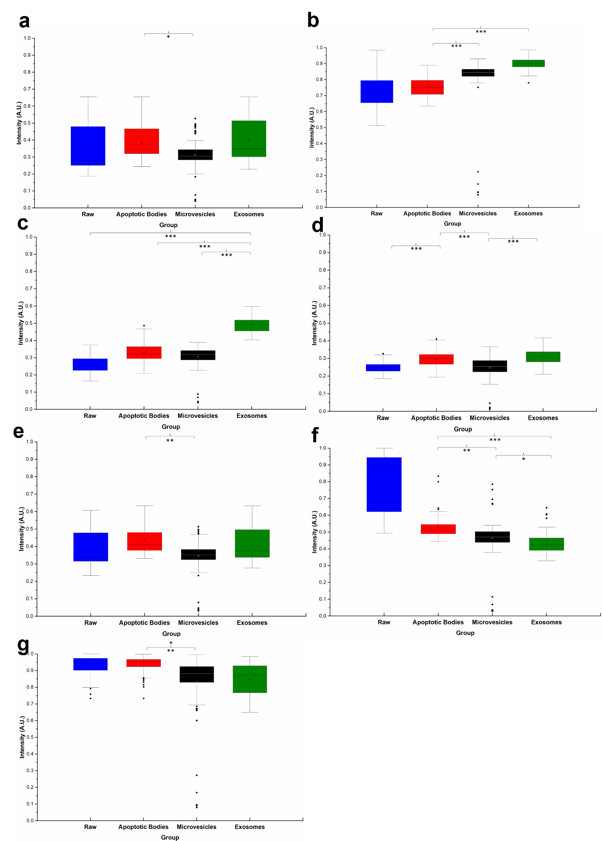


**Figure S6. Spectral Variability Within Healthy Control Blood Plasma Derived EVs.** Box and whisker plots represent the minima, maxima, interquartile ranges, whiskers and the median in peak intensity distributions at **(a)** 851 cm^-1^, **(b)** 960 cm cm^-1^, **(c)** 1003 cm^-1^, **(d)** 1320 cm^-1^, **(e)** 1336 cm^-1^, **(f)** 1448 cm^-1^ and **(g)** 1657 cm^-1^ for healthy control EV subgroups and raw healthy blood plasma. The box represents the IQR with the median indicated by a line inside the box. Whiskers extend to 1.5 times the IQR, and the diamonds denote outliers beyond the whiskers. Intensity levels were significantly higher between EV subgroups and raw plasma. (*p* <0.05, ***p* <0.01 and ****p*<0.0001, student’s *t*-test).

**Table S1.** Change (%) in CVD-indicative biomarkers in raw blood plasma at significant Raman shifts.

| **Marker** | **Raw Plasma (Raman Shift / cm^-1^)** | | | | | |
| --- | --- | --- | --- | --- | --- | --- |
|  | **812** | **1003** | **1296** | **1315** | **1448** | **1656** |
| **IL-9** | 34.1 | 13.4 | 16.7 | 72.2 | 271.4 | 23.5 |
| **ApoB** | 173.3 | 15.8 | 106.7 | 266.7 | 0.02 | 20.1 |
| **PCSK9** | 55.9 | 24 | 42.3 | 23.5 | 69.6 | 140 |
| **LpA** | 10.1 | 24 | 59.4 | 34.1 | 51 | 27.2 |
| **NT-ProBNP** | 60 | 0.03 | 54.5 | 42.6 | 10.3 | 11.6 |

**Table S2.** Change (%) in CVD-indicative biomarkers in apoptotic bodies derived from blood plasma at significant Raman shifts.

| **Marker** | **Apoptotic Bodies (Raman Shift / cm^-1^)** | | | | | |
| --- | --- | --- | --- | --- | --- | --- |
|  | **812** | **1003** | **1296** | **1315** | **1448** | **1656** |
| **IL-9** | 17.4 | 37.3 | 0.06 | 66 | 338 | 0.03 |
| **ApoB** | 78.2 | 43.8 | 38.7 | 0.07 | 19.5 | 20.7 |
| **PCSK9** | 32.3 | 31.4 | 36.8 | 14.2 | 100 | 25 |
| **LpA** | 33.6 | 17.9 | 42.4 | 29.8 | 31.4 | 62.8 |
| **NT-ProBNP** | 82.6 | 17.5 | 24.6 | 29.8 | 31.4 | 62.8 |

**Table S3.** Change (%) in CVD-indicative biomarkers in microvesicles derived from blood plasma at significant Raman shifts.

| **Marker** | **Microvesicles (Raman Shift / cm^-1^)** | | | | | |
| --- | --- | --- | --- | --- | --- | --- |
|  | **812** | **1003** | **1296** | **1315** | **1448** | **1656** |
| **IL-9** | 18.5 | 41.8 | 0.06 | 66 | 338 | 0.03 |
| **ApoB** | 46.3 | 48.4 | 38.7 | 66.7 | 19.5 | 17.1 |
| **PCSK9** | 35.3 | 35.7 | 47.1 | 49.2 | 100 | 250 |
| **LpA** | 35.3 | 167 | 48.6 | 19.5 | 0.06 | 0.06 |
| **NT-ProBNP** | 47.6 | 21.8 | 42.4 | 29.8 | 31.4 | 62.8 |

**Table S4.** Change (%) in CVD-indicative biomarkers in exosomes derived from blood plasma at significant Raman shifts.

| **Marker** | **Exosomes (Raman Shift / cm^-1^)** | | | | | |
| --- | --- | --- | --- | --- | --- | --- |
|  | **812** | **1003** | **1296** | **1315** | **1448** | **1656** |
| **IL-9** | 0.04 | 46.3 | 38.9 | 58.8 | 338 | 23.5 |
| **ApoB** | 31.7 | 53.1 | 19.4 | 59.6 | 22.1 | 10.3 |
| **PCSK9** | 17.6 | 40 | 0.03 | 36.9 | 104 | 160 |
| **LpA** | 17.6 | 165 | 32.4 | 0.03 | 0.04 | 21.2 |
| **NT-ProBNP** | 33.3 | 25.6 | 24.2 | 14.9 | 34.3 | 20.9 |
